# Evaluating the Impact of Robot-Assisted Laparoscopic Pyeloplasty (RALP) on Pediatric Hydronephrosis with and Without Abdominal Pain Symptoms: A Cohort Study Using Inverse Probability of Treatment Weighting (IPTW)

**DOI:** 10.3390/jcm15114347

**Published:** 2026-06-04

**Authors:** Yijun Zhao, Fan Yang, Linfeng Zhu, Wenchang Huang, Xiaohao Wang, Xiang Yan, Guangjie Chen

**Affiliations:** Department of Urology, Children’s Hospital, Zhejiang University School of Medicine, National Clinical Research Center for Children and Adolescents’ Health and Diseases, No. 3333 Binsheng Road, Hangzhou 310003, China; zhaoyj2012@zju.edu.cn (Y.Z.); yangfan1155@zju.edu.cn (F.Y.); zhulf@zju.edu.cn (L.Z.); 22118500@zju.edu.cn (W.H.); ywyywxh@zju.edu.cn (X.W.); yanxiang@zju.edu.cn (X.Y.)

**Keywords:** robot-assisted laparoscopic pyeloplasty, hydronephrosis, symptom, children, inverse probability of treatment weighting

## Abstract

**Objective**: This study aimed to evaluate the effectiveness of robot-assisted laparoscopic pyeloplasty (RALP) in pediatric hydronephrosis with or without abdominal pain symptoms, and to compare post-operative outcomes between these two clinical subtypes. **Methods:** We retrospectively reviewed pediatric patients who underwent RALP at our institution between April 2020 and October 2024. Patients were categorized into hydronephrosis with and without abdominal pain groups. The primary endpoints were renal pelvis anteroposterior diameter (APD) and percent improvement in APD (PI-APD). Inverse probability of treatment weighting (IPTW) was applied to minimize selection bias and balance baseline covariates. **Results:** A total of 273 pediatric patients were analyzed, including 104 cases with abdominal pain and 169 without. After IPTW adjustment, the baseline characteristics of the groups were comparable. At 6 months post-operative, the abdominal pain group showed slower reduction in APD (1.3 cm vs. 1.2 cm, *p* < 0.05) and PI-APD (37% vs. 50%, *p* < 0.05). No significant differences were observed at the 12-month or last follow-up. The overall surgical success rate was 97.1% (265/273). The IPTW-adjusted analysis revealed a higher surgical success rate in the abdominal pain group (99.9% vs. 97.1%, *p* < 0.05). **Conclusions:** RALP is safe and effective for pediatric hydronephrosis, regardless of the presence of abdominal pain. Although both groups showed comparable recovery at 12 months or last follow-up, the patients with abdominal pain exhibited slower improvement at 6 months, suggesting potential differences in early post-operative remodeling between these subtypes.

## 1. Introduction

Hydronephrosis is defined as dilation of the renal collecting system caused by urinary stasis, most commonly resulting from impaired urine outflow from the kidney [1]. Ureteropelvic junction obstruction (UPJO) is the leading cause of hydronephrosis in pediatric patients, with an incidence of approximately 1 in 1500 newborns [2,3]. The clinical spectrum of UPJO is heterogeneous, ranging from asymptomatic cases to symptomatic obstruction. If left untreated, persistent obstruction may lead to progressive renal damage and ultimately renal insufficiency or even renal failure [4]. Therefore, the primary goal of UPJO management is to relieve obstruction and preserve renal function [5].

Hydronephrosis presents with diverse clinical manifestations. Acute obstruction may lead to episodic abdominal or flank pain, often accompanied by nausea or vomiting, a condition known as Dietl’s crisis [6]. Previous studies have demonstrated that the pathological characteristics of the ureteropelvic junction (UPJ) differ between UPJO patients and normal individuals, as well as among hydronephrosis patients with different clinical manifestations [3,7,8]. These variations in pathophysiological and clinical features may influence surgical indications, timing, and post-operative recovery, highlighting the importance of distinguishing these subtypes when evaluating treatment outcomes. However, most clinical studies on pyeloplasty do not differentiate between hydronephrosis with different clinical manifestations, even in the era of robotic surgery [5,9,10]. Dismembered pyeloplasty remains the gold standard for treating UPJO. Compared with open surgery, robot-assisted laparoscopic pyeloplasty (RALP) shares the benefits of conventional laparoscopy (shorter hospital stay, better cosmesis, less post-operative pain, and faster recovery) while also providing a 3D surgical view and highly flexible robotic arms. RALP has emerged as a widely adopted minimally invasive approach in pediatric patients [11].

In the present study, inverse probability of treatment weighting (IPTW) was applied to balance baseline characteristics between hydronephrosis patients with and without abdominal pain, enabling a more rigorous comparison of effectiveness and post-operative pelvic recovery after RALP to inform clinical decision making and follow-up strategies in pediatric UPJO management.

## 2. Patients and Methods

### 2.1. Study Design

We conducted a retrospective analysis of pediatric patients who underwent RALP at our institution between April 2020 and October 2024. Patients were classified into two groups according to the presence or absence of abdominal pain symptoms associated with hydronephrosis.

Pre-operative imaging, including renal ultrasonography and magnetic resonance urography (MRU), was performed to confirm the diagnosis of UPJO and assess the anteroposterior diameter (APD) of the renal pelvis, parenchymal thickness, and split renal function. The diagnosis of UPJO was based on renal ultrasonography or MRU findings of pelvic dilation caused by ureteropelvic junction obstruction, or on a diuretic renography showing a half-time (T½) > 20 min. All standardized Anderson–Hynes dismembered pyeloplasty procedures were performed by experienced surgeons using the da Vinci Xi surgical system. This study was approved by the Ethics Committee of the Children’s Hospital of Zhejiang University School of Medicine (Approval Number: 2025-IRB-0524-P-01).

### 2.2. Study Population

**Figure 1:**
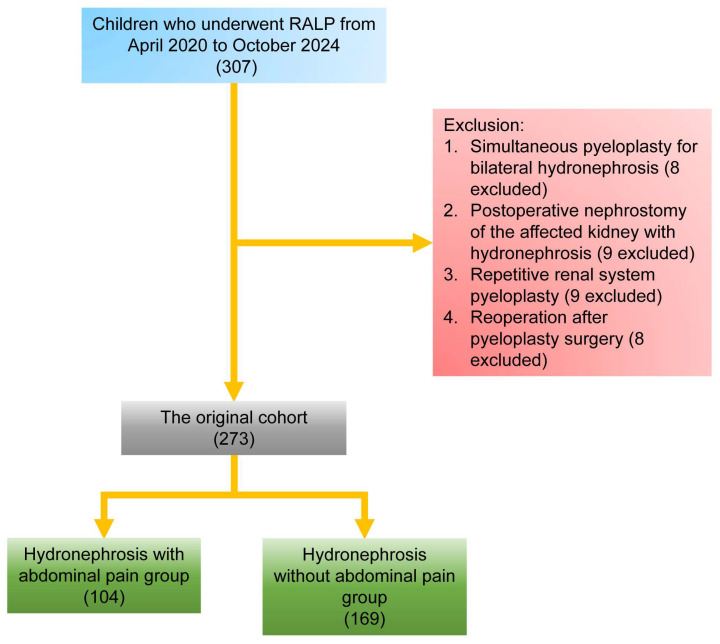
Patient selection strategy.

This retrospective cohort study included patients who were diagnosed with UPJO. The indications for surgery included at least one of the following: (1) hydronephrosis-related clinical symptoms, such as abdominal pain, vomiting, or urinary tract infection; (2) progressive worsening of hydronephrosis on ultrasound follow-up; (3) grade III or IV hydronephrosis according to the Society for Fetal Urology (SFU) classification; or (4) a split renal function < 40% or a decline in split renal function of greater than 5–10% during follow-up. Patients were excluded if they met any of the following criteria: (1) previous ipsilateral pyeloplasty; (2) bilateral hydronephrosis requiring simultaneous pyeloplasty; (3) recurrent hydronephrosis or associated renal abnormalities (e.g., duplex kidney, ectopic kidney, or horseshoe kidney) or malignancies; (4) history of nephrostomy for severe hydronephrosis; or (5) secondary hydronephrosis caused by other conditions, such as urinary tract stones, ureterovesical junction obstruction, or vesicoureteral reflux. The patient enrollment flowchart is shown in Figure 1. A total of 273 individuals were included and subsequently categorized into two groups based on their main symptoms (Figure 1).

### 2.3. Data Collection

Data were extracted from electronic medical records, including age, weight, sex, laterality, symptoms, etiology of UPJO, minimal pre-operative renal parenchymal thickness, pre-operative split renal function, pre-operative APD, surgical time, intra-operative blood loss, and stent size. Age, weight, sex, laterality, and symptoms were collected from the history of present illness. Patients with documented intermittent or persistent abdominal pain related to hydronephrosis before surgery were assigned to the abdominal pain group. Renal parenchymal thickness and APD were measured by renal ultrasonography. Intrinsic stenosis was defined as an intrinsic narrowing of the ureteropelvic junction that was identified intra-operatively as the cause of the UPJO. Surgical time, intra-operative blood loss, and stent size were collected from surgery records. Split renal function was measured by 99mTc-DTPA (Hangzhou Atom High-Tech Medicine Co., Ltd., Hangzhou, China) renal scintigraphy. All the imaging assessments followed a standardized protocol.

### 2.4. Surgical Techniques

All procedures were performed using the da Vinci Xi robotic system (Intuitive Surgical, Inc., Sunnyvale, CA, USA) via a transperitoneal approach. After endotracheal intubation under general anesthesia, the patients were positioned in a 45–80° lateral decubitus position with the healthy side down. An 8 mm camera port was inserted at the umbilicus to establish pneumoperitoneum, with the insufflation pressure adjusted according to patient age. Two additional 8 mm working ports were placed on each side of the midline, and a 5 mm assistant port was placed on the healthy side. Anderson–Hynes dismembered pyeloplasty was then performed, with the placement of an appropriately sized double-J ureteral stent according to the intra-operative findings.

### 2.5. Outcomes

The primary outcomes were APD and percent improvement in APD (PI-APD), which was calculated as follows: PI-APD = (pre-operative APD − post-operative APD)/pre-operative APD × 100. Secondary outcomes were surgical success, surgical complications, duration of drainage tube indwelling, duration of indwelling urinary catheter, duration of indwelling stent, hospitalization expenses, and duration of post-operative hospital stay. Post-operative complications were classified according to the Clavien–Dindo grading system. Post-operative hydronephrosis was assessed through ultrasonography. Surgical success was defined as (1) resolution of pre-operative symptoms (e.g., ipsilateral abdominal pain, hematuria, and urinary tract infection) and (2) improvement of hydronephrosis on consecutive post-operative ultrasonography, defined as a decrease in APD, an increase in PI-APD, and increased parenchymal thickness. Patients were followed for at least 12 months. Outcomes at 6 months, 12 months, and the last follow-up were recorded and compared with the pre-operative values.

### 2.6. Statistical Analysis

Continuous variables were assessed using the Anderson–Darling test and are presented as medians [interquartile ranges (IQRs)]. Categorical variables were expressed as counts and percentages. The Mann–Whitney U and χ^2^ tests were used when appropriate. For analyses in the IPTW-weighted cohort, all tests were conducted using weighted methods to account for the IPTW-derived weights.

IPTW was applied to generate weighted groupings, specifically the weighted cohort, in conjunction with the original cohort. Propensity scores were estimated using a multivariable logistic regression model that included clinically relevant covariates known to influence surgical outcomes: age, weight, sex, laterality, causes of UPJO, pre-operative renal parenchymal thickness, pre-operative split renal function, pre-operative APD, surgical time, intra-operative blood loss, and stent size. This selection ensured adjustment for key potential confounders. Because intra-operative variables could be influenced by pre-operative symptom status and might lie on the causal pathway, we also conducted a sensitivity analysis using a separate IPTW model that included pre-operative covariates only (excluding intrinsic stenosis, surgical time, blood loss, and stent size). This sensitivity analysis was performed to assess whether our primary findings were robust to the inclusion of intra-operative variables. Weights were trimmed to the 1st to 99th percentiles to minimize influence from extreme values. To assess the stability of the IPTW analysis, we examined the distribution of the weights, the degree of overlap between the treated and untreated groups, and calculated the effective sample size (ESS) after weighting. The effectiveness of IPTW was evaluated by calculating standardized mean differences (SMDs). The SMD plots offered a clear and interpretable method for assessing covariate balance between patients with and without abdominal pain. Since the SMD is independent of the unit of measurement, it enables comparisons across variables with different scales, making it especially useful for visual diagnostics [12]. An SMD of less than 0.1 is seen as a reasonable difference between groups [13].

Missing data were assessed for all variables. Variables with missing values were handled using complete-case analysis, which excluded observations with missing values from the relevant analyses. No imputation was performed. To account for multiple comparisons across different follow-up time points, p-values were adjusted using the Bonferroni correction method.

Statistical analysis was conducted using R software (version 4.1.1), with a *p* < 0.05 considered statistically significant.

## 3. Results

### 3.1. Baseline Characteristics

A total of 273 pediatric patients who underwent RALP for UPJO between April 2020 and October 2024 were included in the final analysis. The overall cohort had a median age of 52 (11, 89) months and a median weight of 17.5 (9.8, 24.0) kg. Most patients were male (80.2%), and had left-sided UPJO involvement (78.8%). Abdominal pain was present in 104 patients (38.1%), whereas 169 patients (61.9%) presented without abdominal pain symptoms.

The median minimal pre-operative renal parenchymal thickness was 0.46 (0.30, 0.80) cm, the median split renal function was 43 (35, 47) %, and median pre-operative APD was 2.3 (1.7, 3.1) cm. Intrinsic stenosis was identified intra-operatively in 238 patients (87.2%). The median operative time was 85 (70, 110) min, with minimal blood loss across the cohort. Detailed baseline and perioperative characteristics are summarized in Table 1.

Overall surgical outcomes were favorable. During a median follow-up period of 22 (12, 34) months, the median APD progressively decreased from 2.3 cm pre-operatively to 1.20 cm at 6 months, 1.00 cm at 12 months, and 0.9 cm at the last follow-up. Correspondingly, median PI-APD improved to 48%, 56%, and 59% at the respective follow-up time points. The overall surgical success rate was 97.1%, whereas post-operative complications occurred in only 2.9% of patients.

### 3.2. Baseline Differences Between Groups and IPTW Adjustment

Before weighting, substantial baseline differences were observed between the abdominal pain and no-abdominal pain groups (Table 2). Patients presenting with abdominal pain were significantly older (median 85.5 vs. 24.0 months; SMD = 1.382) and heavier (median 23.0 vs. 12.4 kg; SMD = 1.025) than patients without abdominal pain. In addition, the abdominal pain group had a thicker renal parenchyma (0.70 vs. 0.40 cm; SMD = 0.912), smaller pre-operative APD (1.8 vs. 2.6 cm; SMD = 0.401), and larger stent size (5.0 vs. 4.0 Fr; SMD = 0.975). These findings suggested marked clinical heterogeneity between groups prior to adjustment.

To reduce potential selection bias and improve comparability between groups, IPTW based on propensity scores was applied. Because IPTW generates weighted pseudo-populations rather than patient counts, the weighted sample sizes shown in Table 3 represent the sum of weights rather than true integer patient numbers.

**Figure 2:**
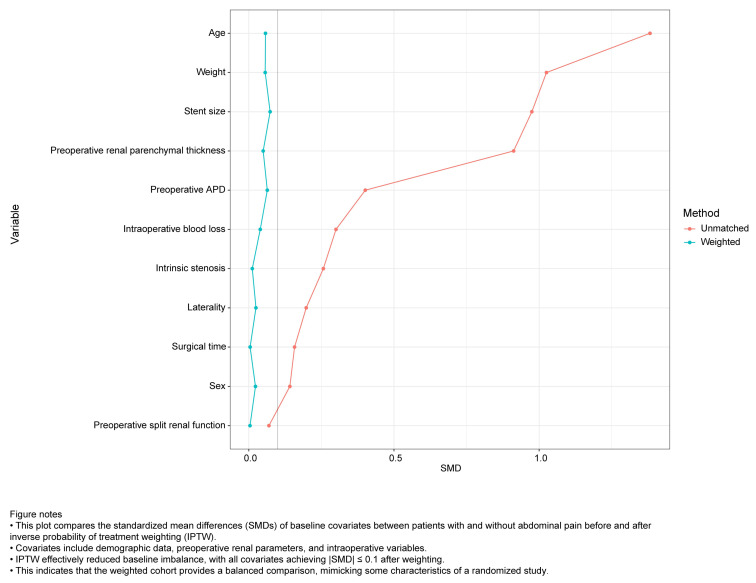
IPTW-adjusted analysis of hydronephrosis with and without abdominal pain groups.

The stability of the IPTW model was further evaluated by examining weight distribution and propensity score overlap (Supplementary Table S1 and Supplementary Figure S1). Before trimming, the overall IPTW weights ranged from 0.118 to 4.216, indicating the presence of several relatively extreme observations. After trimming at the 1st and 99th percentiles, the weight range narrowed to 0.132–2.641, and only six observations were affected, suggesting limited influence of extreme weights on the final model. Propensity score distributions demonstrated substantial overlap between the abdominal pain and no-abdominal pain groups (abdominal pain: 0.067–0.872; no-abdominal pain: 0.045–0.812), supporting the positivity assumption and indicating adequate comparability between groups after weighting by trimming. In addition, the effective sample sizes after trimming were 48.7 and 45.9 in the abdominal pain and no-abdominal pain groups, respectively, indicating acceptable stability of the weighted cohort. After IPTW adjustment, all baseline covariates achieved adequate balance, with the maximum SMD decreasing from 1.382 before weighting to 0.073 after weighting, and no covariate had an SMD greater than 0.1 (Table 3; Figure 2), indicating that IPTW effectively minimized baseline imbalance between groups.

To further evaluate the robustness of the weighting strategy, a sensitivity analysis using only pre-operative covariates was performed. In this analysis, weighted sample sizes were 55.3 and 52.2 in the abdominal pain and no-abdominal pain groups, respectively. All pre-operative covariates remained well balanced, with a maximum SMD of 0.046, confirming that the primary IPTW findings were not substantially influenced by inclusion of intra-operative variables (Supplementary Table S2).

### 3.3. Primary and Secondary Outcomes in Different Cohorts

In the original cohort, patients with abdominal pain showed significantly lower PI-APD values at all follow-up time points compared with the no-abdominal pain group, including at 6 months (42% vs. 50%, *p* = 0.010), 12 months (46% vs. 59%, *p* = 0.025), and the last follow-up (49% vs. 65%, *p* = 0.002) (Table 4). However, post-operative APD values were comparable between groups throughout follow-up.

No statistically significant differences were observed in surgical success rate, post-operative complications, drainage tube duration, urinary catheter duration, stent duration, hospitalization cost, or post-operative hospital stay between the two groups in the unadjusted cohort.

After IPTW adjustment, early post-operative differences remained evident at 6 months. Specifically, patients with abdominal pain group had a slightly larger APD (1.3 cm vs. 1.2 cm, *p* = 0.023) and a lower PI-APD (37% vs. 50%, *p* = 0.002) compared with patients without abdominal pain (Table 5). However, these differences gradually diminished over time. At 12 months and at the last follow-up, no statistically significant differences in APD or PI-APD were identified between groups, suggesting comparable mid- to long-term radiographic improvement after RALP regardless of pre-operative abdominal pain status.

The weighted analysis also demonstrated a slightly higher surgical success rate in the abdominal pain group (99.9% vs. 97.1%, *p* < 0.05). Nevertheless, complication rates and other perioperative recovery indicators, including drainage duration, catheter duration, stent duration, hospitalization costs, and post-operative hospital stay, remained similar between groups after adjustment.

The sensitivity analysis based solely on pre-operative covariates yielded findings consistent with the primary IPTW model. After weighting, the abdominal pain group continued to demonstrate a larger APD (1.3 cm vs. 1.0 cm, *p* = 0.032) and lower PI-APD (52% vs. 63%, *p* = 0.029) at 6 months, whereas no significant differences were observed at later follow-up time points. The surgical success rate also remained higher in the abdominal pain group (99.8% vs. 96.7%, *p* = 0.003). These findings further support the robustness of the primary IPTW analysis (Supplementary Tables S2 and S3).

## 4. Discussion

Hydronephrosis is a common disease in pediatric urology. Its clinical symptoms often include abdominal pain, abdominal masses, and urinary tract infections. However, most patients present with asymptomatic hydronephrosis detected through imaging. The severity of hydronephrosis typically correlates with the degree of renal pelvic dilation [14]. During Dietl’s crisis, the degree of hydronephrosis may increase acutely and then decrease with pain relief. Previous studies have suggested that, when a comprehensive history is taken, hydronephrosis should be suspected in any preschool child with recurrent abdominal pain [15]. With our improved understanding of hydronephrosis, missed diagnoses of hydronephrosis in children presenting with abdominal pain have become increasingly rare. Abdominal pain is a clear surgical indication for treating hydronephrosis. With the continuous development of robotic technology, RALP is now considered the new standard for minimally invasive pediatric surgery [11,16]. In order to better investigate the efficacy of pyeloplasty in patients with different clinical phenotypes of hydronephrosis, we classified children with hydronephrosis treated with RALP at our center based on presence or absence of abdominal pain and performed a comprehensive evaluation.

The study cohort comprised 273 pediatric patients, who represent a broad range of ages, symptom profiles, and disease severities. Hydronephrosis can be diagnosed as early as the prenatal period, and surgical intervention can be performed during infancy, adolescence, or even adulthood. In this study, patients with abdominal pain were on average 61.5 months older (85.5 m vs. 24.0 m) than patients without, which is generally consistent with the previously reported age difference of 60 months [8]. Previous pathological studies have shown that the cellular content of UPJ tissue is related to age, which correlates with post-operative recovery [17,18,19]. Therefore, this baseline diversity (e.g., age) necessitated rigorous statistical control when comparing outcomes between patients with and without abdominal pain. The implementation of IPTW is a major methodological strength of this study, as it enabled adjustment for potential confounders and minimized bias arising from baseline imbalances [9,20]. By generating a weighted population with well-balanced covariates, we were able to more accurately isolate the influence of the hydronephrosis pattern on surgical outcomes, thereby improving the robustness and clinical applicability of our findings [21]. In the original cohort, no statistically significant differences in APD were found after 6 months, 12 months, or at the last follow-up between the two groups, whereas the PI-APD was lower in the abdominal pain group. These differences can be attributed to the difference in pre-operative APD between the groups. Following IPTW adjustment, the baseline characteristics were nearly the same between the groups, especially in terms of age, weight, and pre-operative APD. At 6 months post-operative, the abdominal pain group had a greater APD (1.3 cm vs. 1.2 cm; *p* < 0.05) and a lower PI-APD (0.37 vs. 0.50; *p* < 0.05) than the group without abdominal pain. However, these significant differences in APD and PI-APD disappeared at 12 months post-operative and at the last follow-up. These findings suggest that early morphological improvement of the renal pelvis after pyeloplasty is relatively slow in patients with abdominal pain. However, this disparity was no longer present at 12 months or later, indicating convergence in pelvic remodeling between the two groups over the long term.

Pathological studies have shown that patients with a lower collagen-to-smooth muscle ratio in the UPJ, as well as a lower percentage of elastin in the renal pelvis, UPJ, and ureter, exhibit better improvement after pyeloplasty [18,19]. However, these histopathological patterns do not affect the surgical success rate of pyeloplasty [22]. Apart from pathology, an age of more than 2 years and grade IV hydronephrosis can also lead to slower improvement in the renal pelvis [23]. Although we revealed slower renal pelvis remodeling in the early post-operative period in hydronephrosis with abdominal pain, the underlying mechanism requires further pathological studies. Moreover, our findings suggest that the presence or absence of abdominal pain in hydronephrosis may influence pyeloplasty outcomes.

The overall surgical success rate of 97.1% aligns with previously reported high success rates of RALP in pediatric populations [9,24]. Notably, after IPTW adjustment, a significant difference in surgical success was observed between the groups with and without abdominal pain (99.9% vs. 97.1%, *p* < 0.05). This higher success rate for symptomatic hydronephrosis than for asymptomatic hydronephrosis has also been reported for laparoscopic pyeloplasty [25]. The surgical success criteria state that patients with abdominal pain are considered cured if their pain resolved post-operatively, even if hydronephrosis persisted. This may overestimate the success rate. Extending the follow-up period or performing renal scintigraphy may facilitate the identification of cases falsely classified as successful surgeries. The worse resolution of hydronephrosis but higher rate of surgical success in the abdominal pain group suggests that the detection of surgical failure is also a complex task in unimproved hydronephrosis, especially in the early post-operative phase, and does not necessarily mean failure and warrants a redo procedure [10].

A complication rate of 2.9% was observed, which is slightly lower than the rate of 3.9% that was previously reported in pediatric populations [26]. The low complication rate further confirms the safety of this technique. A total of eight complications were recorded. According to the Clavien–Dindo grading system, five were classified as grade I–II and three as grade IIIb. The abdominal pain group had one grade II complication (post-operative bleeding requiring blood transfusion) and two grade IIIb complications (one case of stent migration and one case of stent obstruction, both requiring stent replacement). In the non-abdominal pain group, there were three grade I complications (two cases of intrapelvic blood clots and one case of perirenal hematoma), one grade II complication (post-operative bleeding requiring blood transfusion), and one grade IIIb complication (post-operative active bleeding from the trocar incision requiring laparoscopic exploration). Although the complication rate of RALP is lower than that of laparoscopic pyeloplasty (LP), the single case of trocar-site bleeding warrants careful attention. This suggests that we should be vigilant for abdominal wall vascular injury during trocar placement.

In addition, we examined several secondary endpoints relevant to patient recovery and healthcare utilization, including drainage tube duration, catheter duration, stent indwelling time, hospitalization costs, and post-operative hospital stay. Although no difference was found, these indicators provide important insight into the procedural burden on both patients and medical systems.

Several limitations merit consideration. First, the retrospective design introduces the possibility of selection bias, such as potential symptom misclassification, subjective outcome definitions, and unidentified surgical failure cases, despite comprehensive statistical adjustment. Second, the single-center nature of the study may restrict its external validity. Prospective, multicenter investigations with longer follow-up periods are needed to corroborate and extend the observations in this study. Third, pathological studies should be conducted on the mechanism of renal pelvis remodeling.

In summary, this retrospective observational study suggests that, after applying IPTW to address pre-operative heterogeneity, RALP achieves comparable outcomes in children with and without abdominal pain. Although the children with abdominal pain exhibited slower early morphological recovery, satisfactory improvement was evident by 12 months and was sustained at the last follow-up. These findings suggest that the presence of abdominal pain may affect early recovery after RALP but not overall RALP efficacy and safety.

## Figures and Tables

**Table 1:** Demographic and clinical features of the whole cohort.

Covariate	Value
N	273
Baseline characteristics	
Age (months)	52 (11, 89)
Weight (kg)	17.5 (9.8, 24.0)
Sex	
Male	219 (80.2%)
Female	54 (19.8%)
Laterality	
Left	215 (78.8%)
Right	58 (21.2%)
Symptom	
Yes	104 (38.1%)
No	169 (61.9%)
Minimal pre-operative renal parenchymal thickness (cm)	0.46 (0.30, 0.80)
Pre-operative split renal function (%)	43 (35, 47)
Pre-operative APD (cm)	2.3 (1.7, 3.1)
Intra-operative variables	
Intrinsic stenosis	
Yes	238 (87.2%)
No	35 (12.8%)
Surgical time (min)	85 (70, 110)
Intra-operative blood loss (mL)	2 (2, 2)
Stent size (Fr)	4.8 (4.0, 5.0)
Post-operative outcomes	
6-month post-operative APD (cm)	1.20 (0.85, 1.60)
6-month post-operative PI-APD (%)	48 (29, 64)
12-month post-operative APD (cm)	1.00 (0.72, 1.40)
12-month post-operative PI-APD (%)	56 (36, 68)
Last follow-up APD (cm)	0.9 (0.6, 1.3)
Last follow-up PI-APD (%)	59 (39, 77)
Surgical success (%)	
Yes	265 (97.1%)
No	8 (2.9%)
Surgical complication (%)	
Yes	8 (2.9%)
No	265 (97.1%)
Duration of drainage tube (days)	3 (3, 4)
Duration of indwelling urinary catheter (days)	6 (5, 6)
Duration of indwelling stent (days)	38 (34, 45)
Hospitalization cost (yuan)	62,811 (61,278, 64,961)
Post-operative hospital stay (days)	6 (6, 7)

**Table 2:** Demographic and clinical features of the original cohort.

Covariate	Hydronephrosis with Abdominal Pain	Hydronephrosis Without Abdominal Pain	*SMD*
N	104	169	
Baseline characteristics			
Age (months)	85.5 (65.0, 107.5)	24 (5.5, 58)	1.382
Weight (kg)	23 (19, 30.1)	12.4 (7.5, 19.0)	1.025
Sex			0.141
Male	87 (83.7%)	132 (78.1%)	
Female	17 (16.3%)	37 (21.9%)	
Laterality			0.198
Left	87 (83.7%)	128 (75.7%)	
Right	17 (16.3%)	41 (24.3%)	
Minimal pre-operative renal parenchymal thickness (cm)	0.70 (0.49, 1.10)	0.40 (0.27, 0.56)	0.912
Pre-operative split renal function (%)	44 (36, 48)	41 (34, 47)	0.069
Pre-operative APD (cm)	1.8 (1.3, 2.7)	2.6 (2.0, 3.3)	0.401
Intra-operative variables			
Intrinsic stenosis			0.257
Yes	85 (81.7%)	153 (90.5%)	
No	19 (18.3%)	16 (9.5%)	
Surgical time (min)	90 (70, 120)	85 (70, 110)	0.158
Intra-operative blood loss (mL)	2 (2, 2)	2 (2, 2)	0.300
Stent size (Fr)	5.0 (4.8, 5.0)	4.0 (4.0, 4.8)	0.975

**Table 3:** Demographic and clinical features of the weighted cohort.

Covariate	Hydronephrosis with Abdominal Pain	Hydronephrosis Without Abdominal Pain	*SMD*
N	56.8	53.8	
Baseline characteristics			
Age (months)	69.25 (52.08, 87.00)	76.00 (43.10, 96.87)	0.057
Weight (kg)	20.09 (17.04, 25.42)	20.00 (15.63, 26.81)	0.056
Sex			0.023
Male	48.3 (85.0%)	45.3 (84.2%)	
Female	8.5 (15.0%)	8.5 (15.8%)	
Laterality			0.025
Left	47.1 (82.9%)	47 (87.4%)	
Right	9.7 (17.1%)	6.8 (12.6%)	
Minimal pre-operative renal parenchymal thickness (cm)	0.53 (0.40, 0.80)	0.50 (0.35, 0.90)	0.049
Pre-operative split renal function (%)	43 (36, 46)	41 (35, 46)	0.004
Pre-operative APD (cm)	2.03 (1.30, 3.69)	2.40 (2.00, 3.30)	0.064
Intra-operative variables			
Intrinsic stenosis			0.012
Yes	49.8 (87.8%)	44.1 (82.0%)	
No	7 (12.2%)	9.7 (18.0%)	
Surgical time (min)	85 (70, 105)	85 (65, 105)	0.004
Intra-operative blood loss (mL)	2 (2, 2)	2 (2, 2)	0.039
Stent size (Fr)	4.80 (4.00, 5.00)	5.00 (4.46, 5.00)	0.073

**Table 4:** Outcomes of the original cohort.

Covariate	Hydronephrosis with Abdominal Pain	Hydronephrosis Without Abdominal Pain	*p*
N	104	169	
6-month post-operative APD (cm)	1.20 (0.80, 1.70)	1.30 (0.95, 1.55)	0.680
6-month post-operative PI-APD (%)	42 (14, 60)	50 (37, 65)	0.010
12-month post-operative APD (cm)	1.00 (0.71, 1.50)	1.00 (0.80, 1.40)	0.738
12-month post-operative PI-APD (%)	46 (23, 70)	59 (42, 68)	0.025
Last follow-up APD (cm)	0.95 (0.68, 1.40)	0.90 (0.60, 1.30)	0.628
Last follow-up PI-APD (%)	49 (20, 73)	65 (45, 78)	0.002
Surgical success (%)			0.161
Yes	103 (99.0%)	162 (95.9%)	
No	1 (1.0%)	7 (4.1%)	
Surgical complication (%)			1.000
Yes	3 (2.9%)	5 (3.0%)	
No	101 (97.1%)	164 (97.0%)	
Duration of drainage tube (days)	3 (3, 4)	3 (3, 4)	0.567
Duration of indwelling urinary catheter (days)	6 (5, 6)	6 (5, 6)	0.826
Duration of indwelling stent (days)	40 (33, 47)	38 (34, 45)	0.273
Hospitalization cost (yuan)	62,628 (61,331, 64,165)	62,819 (61,178, 65,730)	0.362
Post-operative hospital stay (days)	6 (6, 8)	6 (6, 7)	0.086

**Table 5:** Outcomes of the weighted cohort.

Covariate	Hydronephrosis with Abdominal Pain	Hydronephrosis Without Abdominal Pain	*p*
N	56.8	53.8	
6-month post-operative APD (cm)	1.30 (1.11, 1.73)	1.20 (0.97, 1.60)	0.023
6-month post-operative PI-APD (%)	37 (23, 50)	50 (32, 65)	0.002
12-month post-operative APD (cm)	1.00 (0.80, 1.49)	1.20 (0.80, 1.70)	0.318
12-month post-operative PI-APD (%)	50 (22, 72)	51 (35, 68)	0.695
Last follow-up APD (cm)	0.90 (0.66, 1.30)	1.00 (0.60, 1.40)	0.428
Last follow-up PI-APD (%)	50 (38, 56)	56 (41, 73)	0.724
Surgical success (%)			<0.05
Yes	56.7 (99.9%)	52.2 (97.1%)	
No	0.1 (0.1%)	1.6 (2.9%)	
Surgical complication (%)			0.100
Yes	0.9 (1.5%)	3.3 (6.2%)	
No	55.9 (98.5%)	50.5 (93.8%)	
Duration of drainage tube (days)	3 (3, 5)	3 (3, 4)	0.755
Duration of indwelling urinary catheter (days)	6 (5, 7)	6 (5, 6)	0.870
Duration of indwelling stent (days)	39 (33, 47)	31 (33, 47)	0.347
Hospitalization cost (yuan)	63,000 (61,507, 64,203)	61,825 (59,640, 64,646)	0.060
Post-operative hospital stay (days)	6 (6, 7)	6 (6, 7)	0.233

## Data Availability

The datasets used and analyzed in the current study are available from the corresponding author upon reasonable request. The data are not publicly available because of privacy or ethical restrictions.

## Supplementary Materials

The following supporting information can be downloaded at: https://www.mdpi.com/article/10.3390/jcm15114347/s1, Figure S1: Propensity Score Overlap Plot; Table S1: Simulated diagnostics of IPTW weight stability; Table S2: Baseline characteristics of the IPTW-weighted cohort using preoperative covariates only; Table S3: Outcomes of the IPTW-weighted cohort using preoperative covariates only.
