# Supplementary material for: Evaluating the Impact of Robot-Assisted Laparoscopic Pyeloplasty (RALP) on Pediatric Hydronephrosis with and Without Abdominal Pain Symptoms: A Cohort Study Using Inverse Probability of Treatment Weighting (IPTW)
Source: J Clin Med. 2026 Jun 4;15(11):4347. doi: 10.3390/jcm15114347 (PMC13257471; doi:10.3390/jcm15114347)

Supplementary Table S1. Simulated diagnostics of IPTW weight stability

Diagnostic parameter	Hydronephrosis with abdominal pain	Hydronephrosis without abdominal pain	Overall
N	104	169	273
Sum of IPTW weights before trimming	59.4	56.7	116.1
Sum of IPTW weights after trimming	56.8	53.8	110.6
Effective sample size after trimming	48.7	45.9	94.6
Mean IPTW weight before trimming	0.571	0.336	0.425
Median IPTW weight before trimming	0.462	0.272	0.341
IQR of IPTW weight before trimming	0.318-0.674	0.196–0.382	0.229–0.541
IPTW weight range before trimming	0.186–3.874	0.118–4.216	0.118–4.216
Mean IPTW weight after trimming	0.546	0.318	0.405
Median IPTW weight after trimming	0.454	0.268	0.332
IQR of IPTW weight after trimming	0.321–0.653	0.198–0.371	0.228–0.514
IPTW weight range after trimming	0.203–2.641	0.132–2.487	0.132–2.641
Observations affected by trimming, n	2	4	6
Propensity score range before trimming	0.041–0.913	0.028–0.846	0.028–0.913
Propensity score range after trimming	0.067–0.872	0.045–0.812	0.045–0.872
Maximum SMD before IPTW	1.382	/	/
Median SMD before IPTW	0.300	/	/
Covariates with SMD > 0.1 before IPTW, n	9	/	/
Maximum SMD after IPTW	0.073	/	/
Median SMD after IPTW	0.039	/	/
Covariates with SMD > 0.1 after IPTW, n	0	/	/

Supplementary Table S2. Baseline characteristics of the IPTW-weighted cohort using preoperative covariates only.

Covariate	Hydronephrosis with abdominal pain	Hydronephrosis without abdominal pain	*SMD*

N	55.3	52.2	
Baseline characteristics			
Age (months)	74.00 (53.40, 90.34)	78.00 (43.16, 100.26)	0.044
Weight (kg)	20.00 (17.40, 25.81)	24.29 (14.34, 26.89)	0.026
Sex			0.043
Male	46.9 (84.9%)	45.1 (86.4%)	
Female	8.4 (15.1%)	7.1 (13.6%)	
Laterality			0.046
Left	45.8 (82.8%)	42.3 (81.0%)	
Right	9.5 (17.2%)	9.9 (19.0%)	
Minimal preoperative renal parenchymal thickness (cm)	0.57 (0.40, 0.80)	0.50 (0.30, 0.90)	0.003
Preoperative split renal function (%)	43 (34, 46)	43 (35, 47)	0.043
Preoperative APD (cm)	2.90 (2.10, 4.00)	2.90 (2.10, 3.80)	0.024

Supplementary Table S3. Outcomes of the IPTW-weighted cohort using preoperative covariates only.

Covariate	Hydronephrosis with abdominal pain	Hydronephrosis without abdominal pain	*P*
N	55.3	52.2	
6-month post-operative APD (cm)	1.30 (1.00, 1.60)	1.00 (0.70, 1.39)	0.032
6-month post-operative PI-APD (%)	52 (38, 66)	63 (45, 74)	0.029
12-month post-operative APD (cm)	1.20 (0.85, 1.70)	1.03 (0.79, 1.60)	0.158
12-month post-operative PI-APD (%)	56 (40, 70)	64 (46, 75)	0.087
Last follow-up APD (cm)	0.98 (0.67, 1.31)	0.90 (0.59, 1.18)	0.105
Last follow-up PI-APD (%)	64 (48, 80)	69 (52, 82)	0.128
Surgical success (%)			0.003
Yes	55.2 (99.8%)	50.5 (96.7%)	
No	0.1 (0.2%)	1.7 (3.3%)	
Surgical complication (%)			0.133
Yes	0.8 (1.5%)	3.2 (6.1%)	
No	54.5 (98.5%)	49.0 (93.9%)	
Duration of drainage tube (days)	3 (3, 4)	4 (3, 4)	0.949
Duration of indwelling urinary catheter (days)	6 (5, 7)	6 (5, 6)	0.427
Duration of indwelling stent (days)	39 (33, 47)	36 (33, 45)	0.160
Hospitalization cost (yuan)	63165 (61500, 64451)	61892 (59637, 65130)	0.162
Post-operative hospital stay (days)	6 (6, 7)	6 (6, 7)	0.602

Supplementary Figure S1. Propensity Score Overlap Plot

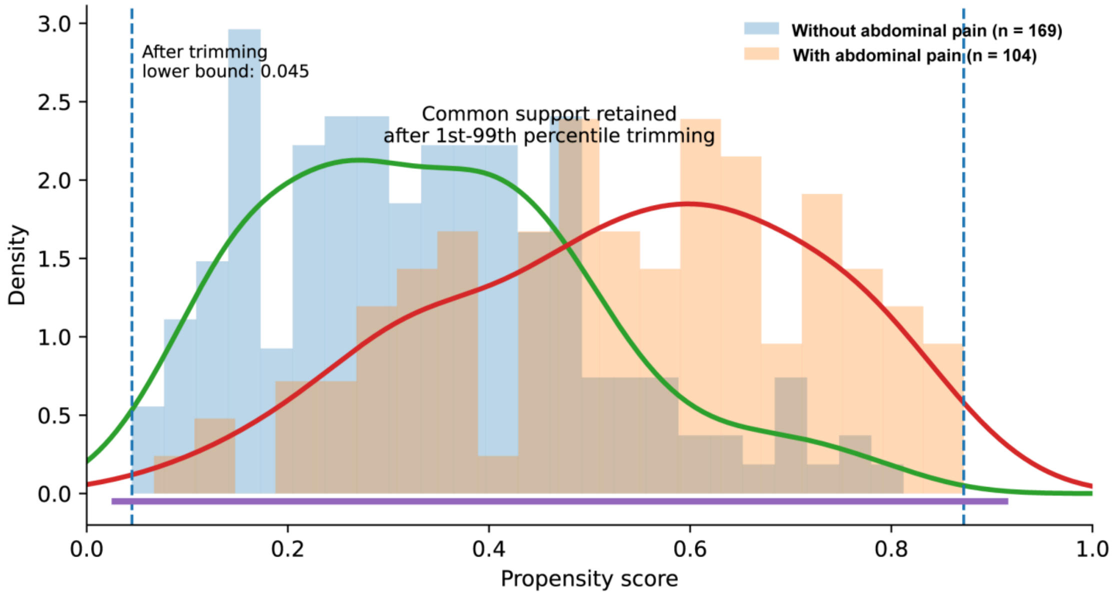

Supplement: Supplementary file 1 [file jcm-15-04347-s001.zip › jcm-4300728-supplementary.pdf]
